# French virtual multidisciplinary team meeting for pediatric movement disorders (PMD-vMDT): a three-year survey

**DOI:** 10.3389/fneur.2025.1751665

**Published:** 2026-01-13

**Authors:** Marie-Céline François-Heude, Bérénice Lecardonnel, Matthildi Papathanasiou, Eline Chauvet-Piat, Cécile Laroche, Marie-Aude Spitz, Jean-Gilles Rodier, Agathe Roubertie, the PMD-vMDT group, Marie Thérèse Abi Warde, Lucile Altenburger, Céline Biboulet Bruneau, Julie Bonheur, Domitille Bommier Laur, Cécile Ians-Bouteiller Ians-Bouteiller, juliette Bouchereau, Lydie Burglen, Marga Buzatu, Sébastien Cabasson, Hugues Chevassus, Mondher Chouchane, Pierre Cleuziou, Arthur Coget, Thibaud Dabudyk, Lena Damaj, Lattre Capucine de, Nathalie Dorison, Diane Doummar, fanny Dubois, Clarisse Gins, Barde Mairena Heidy, Domitille Gras, Paris-Saclay CEA, Marie Hully, Kaoutar Khabbach, Anna Loussouarn, Nicolas Leboucq, Laurence Lion-François, Cyril Mignot, Rebecca More, Chloé Pacteau, Eleni Panagiotakaki, Claudia Ravelli, Florence Riant, Clotilde Rivier Ringenbach, Emmanuel Roze, Thomas Roujeau, Eugénie Sarda, Catherine Sarret, Calina Todosi, Pia Vayssiere, Apo Constance Yapo, Elise Yazbeck

**Affiliations:** 1 Service de Neuropédiatrie, CHU Gui de Chauliac, Montpellier, France; 2 Department of Paediatric Clinical Epileptology, Sleep Disorders and Functional Neurology, University Hospitals of Lyon (HCL), Lyon, France; 3 Service de Pédiatrie, Hôpital mère Enfant, CHU Limoges, France; 4 Service de Neuropédiatrie, Hôpitaux Universitaires de Strasbourg, Strasbourg, France; 5 Service Qualité, CHG Ales-Cevennes, Ales, France; 6 Institut des Neurosciences, INSERM U 1298, Montpellier, France

**Keywords:** multidisciplinary team, patient’s outcome, pediatric movement disorder, survey, virtual MDT

## Abstract

**Introduction:**

Since 2018, a French virtual multidisciplinary team meeting (vMDT), dedicated to discussing the semiological, diagnostical, and therapeutic management of children with movement disorders (MD) has been established, based on medical history and video recordings. Participants included physicians and paramedics involved in the management of pediatric MD (PMD) from France and French-speaking countries. Our aim was to explore how the participants who submitted cases experienced and perceived the process by evaluating overall satisfaction, impact of the PMD-vMDT recommendations on patients’ outcome according to semiological, diagnostic or therapeutic opinion request.

**Materials and methods:**

A qualitative assessment of the PMD-vMDT was carried out using a standardized questionnaire sent to physicians who submitted a case between 2021 and 2024.

**Results:**

We received 83 responses from 46 different practitioners out of 130 cases with requests concerning combined semiological opinion (24 cases), semiological/diagnostic opinion (61 cases), combined therapeutic opinion (65 cases). Semiological categorization of the MD was revised or implemented in 40% of the submitted cases. PMD-vMDT was perceived as helpful for diagnosis decision-making and for etiological workup by 70 and 80% of the respondents, respectively. v PMD-vMDT diagnosis recommendations were implemented by 94% of the respondents, and treatment was modified based on PMD-vMDT in 68% of cases. Overall satisfaction was high, with a mean score of 9.1/10; 89% of the respondents considered that the PMD-vMDT contributed to improvement of their understanding and management of pediatric movement disorders.

**Conclusion:**

This survey highlights the relevance of this pioneering PMD-vMDT for decision-making, patient management, and pedagogical impact.

## 1 Introduction

Multidisciplinary team meetings (MDTs) are defined as “a group of professionals from one or more clinical disciplines who together make decisions regarding recommended treatment of individual patients’ (1). Initially developed in the 90’s, they have become common practise and gold-standard for the care of patients with oncological malignancies. The literature supports the positive impact of MDTs on assessment and management of patients with cancers, and on fostering equitable access to high-quality care (2–4). The concept of virtual MDTs has emerged more recently, with a marked acceleration since the COVID-19 pandemic; virtual MDT have been rapidly expanded to patients with other complex diseases encompassing non-oncological disorders; nevertheless outside oncology, data regarding the assessment of vMDT remains scarce (5–9).

Pediatric Movement disorders (PMD) represent a heterogeneous group of conditions that may be hyperkinetic (tics, stereotypies, dystonia, chorea, athetosis, ballism, tremors, myoclonus) or hypokinetic (parkinsonism). They are frequently *misinterpreted or associated* with other neurological symptoms such as ataxia, epileptic seizures, spasticity, developmental coordination disorders, tone abnormalities, abnormal ocular movements, and behavioral or psychiatric disturbances. Semiological analysis is therefore crucial to guide both etiological diagnosis and therapeutic management. The first step is to confirm or exclude the presence of movement disorders among other neurological symptoms, followed by identification of the specific type of movement disorder. PMD may be isolated or combined, persistent or paroxysmal, which deepen the complexity of their recognition; etiologies of PMD are various, including acquired causes, inherited metabolic diseases, genetic disorders or neurodegenerative processes; a tailored and dedicated diagnosis strategy is necessary to reach a diagnosis. Finally, therapeutic approach may be challenging, as the physiopathology of PMD is not fully understood (10–12). Therefore careful and expert assessment is crucial to ensure appropriate diagnostic and therapeutic management (13, 14). Many pathologies with PMD are highly rare situations not common in daily practise, and that may represent a caveat to achieve accurate diagnosis and optimal treatment.

To address these needs and improve the management of children with movement disorders, a Virtual French MDT (PMD-vMDT) has been implemented in France since 2018, with an operating charter formalizing its organisation. Sessions gather healthcare professionals including physicians (neuropediatricians, pediatricians involved in PMD, epileptologists, geneticists, pediatricians involved in inherited metabolic diseases, pediatric rehabilitation physicians, pediatric neuroradiologists, neurosurgeons) and rehabilitation professionals (physiotherapists, language or occupational therapists), from France mainly, and also Belgium, Switzerland and French-speaking African countries (Morocco, Algeria, Senegal, Ivory Cost, Benin). Number of participants ranges between 17 and 41 (mean 27), with a permanent panel of at least 4 PMD experts, a core group of 10–15 recurrent participants, and occasional attendees. Each session lasts 2–3 h and takes place every 2–3 months, during which 4–8 cases are submitted for discussion. The objective is the discussion of clinical cases involving movement disorders, based on medical history and video recordings. After obtention of informed consent from the parents/caregivers, the participants who submit a case share medical data (summary of the case on a standardized form, and videos) a couple days before the meeting by uploading them on a secured virtual platform provided by Montpellier University hospital. Suitability of the case and of the data is controlled by the vMDT coordinator (AR) before the session. After the session, a formal report of the discussions including consensual recommendations or recording the various opinions and their justification is prepared and validated by the coordinator of the MDT (AR), then reviewed by at least another member of the expert panel.

The main objective of our study is to conduct a survey of the PMT-vMDT to understand how the participants who submitted cases experienced and perceived the process.

## 2 Materials and methods

A retrospective qualitative assessment of the PMD-vMDT was carried out using a standardized questionnaire sent to professional caregivers who submitted a case between 2021 and 2024. Each questionnaire was related to a submitted case, and questioned the physician regarding this specific vMDT case. Physician could complete as many questionnaires as they had submitted cases to the vMDT. Questionnaires were collected at least 6 months after case submission to the vMDT to obtain of follow-up information.

The questionnaire was elaborated by an experienced qualitative physician (JJR). The questionnaire collected objective and subjective data on professionals’ experience before, during, and after vMDT. The form included 46 items with single-choice items, satisfaction scales (from 1 to 10), and open-ended questions. The questionnaire assessed the functioning and impact of the vMDT across five domains: preparation before the meeting [information to families, ease of submitting a case, type of movement disorders according to established classification (15), nature of the request (semiological opinion, diagnostic opinion, therapeutic advice, in particular deep brain stimulation)]; organisation of the vMDT (schedule adherence, quality of discussions, consensus, report accuracy; immediate post-vMDT impact on diagnosis and patient’s management; long-term follow-up and perceived influence of the vMDT on disease course); and overall satisfaction, including usefulness in practice, educational impact and suggestions for improvement. Detailed informations and the full questionnaire are available in Supplementary material 1.

Data concerning vMDT content were extracted from the pre-vMDT forms and from the final reports.

Data are presented using mean (avg), median (med), standard deviation (*σ*) and the maximum and minimum extreme values in the form avg. ± *σ*, med, [min, max] for continuous variables, and frequencies and proportions (%) for categorical variables.

## 3 Results

### 3.1 Referees-respondents

Among the 130 cases discussed between January 2021 and May 2024 during 22 vMDT sessions, 122 questionnaires were sent to the referring healthcare professionals. Nine forms could not be sent as the referees could not be reached. With a response rate of 71% (87/122), we collected 83 questionnaires suitable for analysis (2 forms did not correspond to the expected patients, and 2 were duplicates). Data concerning satisfactory survey were extracted from 80 questionnaires (three questionnaires were not included as the respondent was the vMDT coordinator).

These responses came from 46 healthcare professionals: 19 neuropediatricians, 2 adult neurologists, 19 pediatricians, 1 pediatric orthopedist, 3 rehabilitation medicine physicians, 1 general practitioner and 1 physiotherapist. The referees practised in 30 different centers, including 27 in France and 3 abroad (Switzerland, Belgium, Morocco). Among these centers, 29 were hospital institutions, including 25 university hospitals and 1 private practice.

### 3.2 Consent and patient’s information

All the professional who responded to the question stated that they informed the family of the vMDT referral (1 not known, NK); one respondent did not inform the family of the video sharing. Respondents reported that vMDT outcome was communicated to families in 51% of cases (43/83, 1 NK), and 84% of reports (70/83, 1 NK) were included to the patients’ medical file.

### 3.3 Conduct of the vMDT

**Figure 1:**
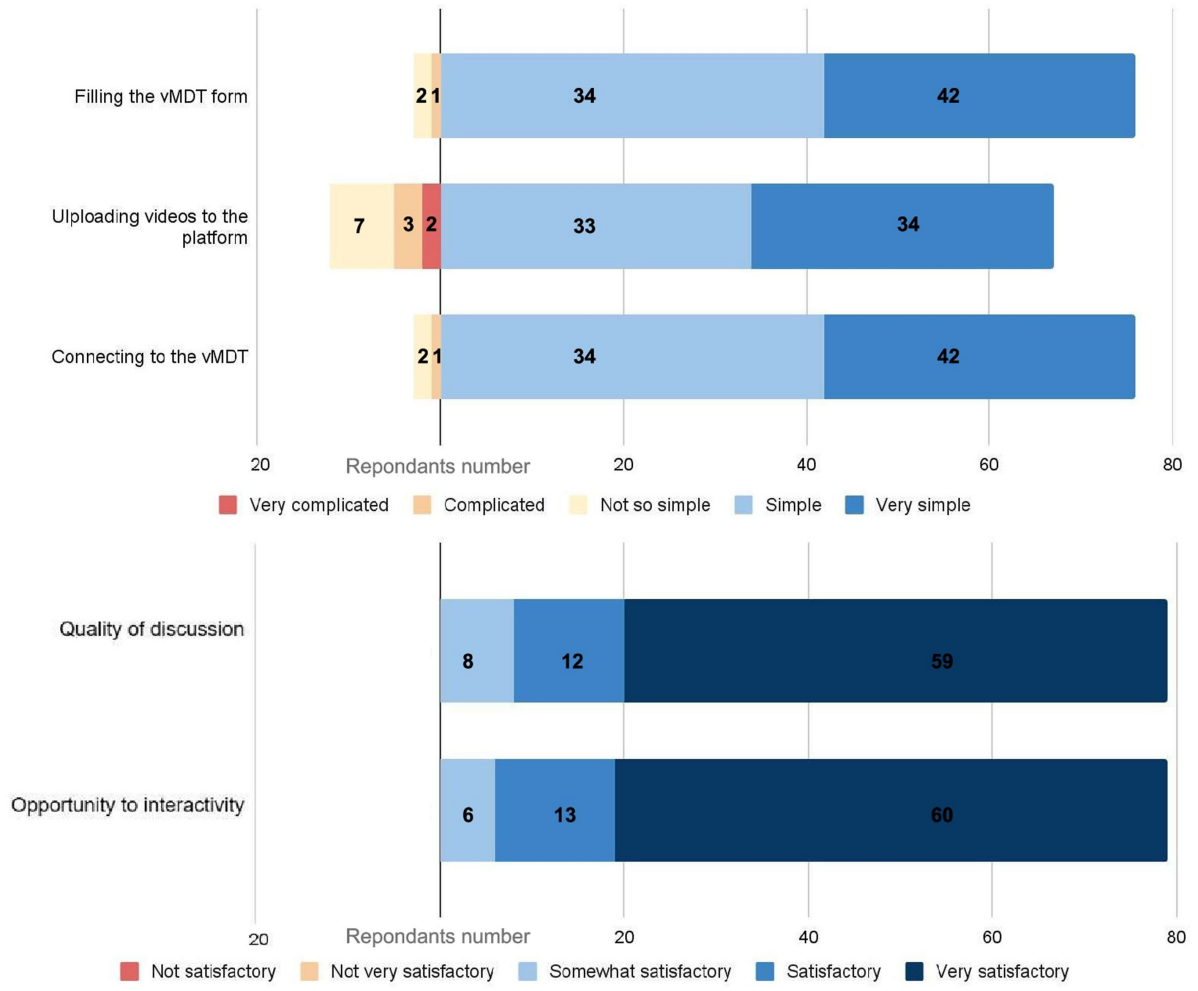
1Evaluation of the virtual multidisciplinary team meeting (vMDT) process and user experience (*n* = 80 *respondents*). The upper panel illustrates respondents’ assessment of the technical aspects of the vMDT. The lower panel presents respondents’ satisfaction regarding the quality of discussions and the opportunities for interactivity during the vMDT.

95% (76/80) of the survey respondents rated technical access to PMD-vMDT as easy for filling MDT standardized form in 95%, and easy for vMDT connexion to the videoconferencing platform; 67/80 were satisfied with the easiness to upload documents and 12 experienced uploading as not so easy to complex (Figure 1).

All respondents except 1 (81/82 (97.5%), 1 NK) stated that the case was reviewed by specialists well trained to the clinical question. All respondents considered positively the quality of discussions or interactivity during vMDT (Figure 1). All participants perceived that their own opinion had been taken into account (1 NK). According to the survey, the final decision reached during the vMDT was consensual in 95.1% (79/82, 1 NK) and considered well-justified by 81/82 respondents (97.5%, 1 NK). Concordance between the vMDT discussions and the report had a mean score of 9.1/10 (7/10 lowest score); 74 respondents considered the report as non-questionable, and 5 considered it as questionable.

### 3.4 Assessment depending on the type of opinion request

In 77 of the 83 cases, the referral to the vMDT represented the first request for an expert opinion; in 6 cases, previous expert opinion had already been solicited.

Respondents requested combined recommendations: combined semiological opinion in 24, semiological/diagnostic opinion in 61 cases, and combined therapeutic opinion in 65 cases. Semiological opinion was in no case an isolated request, an isolated therapeutic recommendation was requested in 22 cases (including 7 concerning DBS). vMDT proposed semiological, diagnostic and/or therapeutic recommendations whenever specifically requested, or extending beyond the scope of the request (for example, semiological opinion was proposed although only therapeutic opinion was requested by the referees in 2 cases; therapeutic advice was proposed when only semiological/diagnostic recommendations were requested). Perceptions by the respondents of the recommendations provided by the vMDT are summarized in Table 1.

**Table 1:** Respondents’ perception of the vMDT recommendations according to the type of request.

vMDT semiological recommendation	Semiological opinion request	Semiological and/or diagnosis opinion request	Only-therapeutic opinion request	Therapeutic + opinion request	All the cases
	*n* = 24	*n* = 61	*n* = 22	*n* = 65	*n* = 83
Revision	1	4	0	2	4
Implementation	11	17	2	15	19
Not modified/NK	11/0	30/10	10/8	28/20	40/20
Was the vMDT helpful for diagnosis decision-making?	19 yes	43 yes	0 yes	27 yes	45 yes
	5 no	8 no	10 no	17 no	19 no
Did the semiological recommendation improved the patient’s outcome?	23 yes	46 yes	6 yes	37 yes	52 yes
	1 no	5 no	5 no	8 no	10 no
Was the vMDT helpful for diagnostic work-up?	23 yes	49 yes	2 yes	34 yes	51 yes
	1 no	12 no	9 no	20 no	21 no
Did you follow the diagnostic recommendations?	24 yes	59 yes	9 yes	51 yes	68 yes
	0 no	2 no	2 no	3 no	4 no
Did the diagnostic recommendation improved the patient’s outcome?	15 yes	35 yes	1 yes	25 yes	36 yes
	4 no	16 no	7 no	9 no	23 no
Were the therapeutic recommendations helpful for decision-making?	21 yes	56 yes	15 yes	50 yes	61 yes
	1 no	9 no	5 no	11no	15 no
Did you follow the therapeutic recommendations?	22 yes	52 yes	17 yes	56 yes	68 yes
	0 no	2 no	3 no	4 no	5 no
Did you change the treatment?	14 yes	32 yes	14 yes	41 yes	46 yes
	8 no	22 no	6 no	19 no	28 no
Did the therapeutic recommendation improved the patient’s outcome?	8 yes	15 yes	5 yes	17 yes	20 yes
	5 no	16 no	8 no	18 no	24 no

Column “semiological opinion request” include semiological + diagnosis requests, semiological + therapeutic requests, and semiological + diagnostic + therapeutic requests (semiological request was never isolated). Column “semiological and/or diagnosis opinion requested" plus “only therapeutic opinion requested" gather all the cases (equal to column “all the cases"). Therapeutic + opinion request column includes therapeutic request, semiological + therapeutic, diagnostic + therapeutic, semiological +diagnosis + therapeutic opinion requests.

According to the respondents, vMDT diagnosis and therapeutic recommendations were implemented by 94 and 93% of the referees, respectively. Among 24 respondents who required a semiological recommendation, 1 reported that the pre-vMDT semiological movement disorder categorisation was revised, 11 reported implementation, and 10 reported no modification. When considering the whole survey, pre-vMDT semiological movement disorder categorisation modified in 36% (revised in 4, implemented in 19, not modified in 40, and not known in 20). The respondents indicated in the survey that the vMDT suggested a genetic diagnosis in 26 cases, which was confirmed in 9, not confirmed in 12 and pending in 5. Among the 61 cases for which a semiological and/or diagnosis opinion was requested, the survey indicated that vMDT recommendations were helpful for diagnosis decision-making in 43/61 (70%) and for etiological workup in 49/61 (80%).

Among cases with a therapeutic opinion request and available response, a treatment change was implemented according to vMDT recommendations 68% (41/60, 5 NK). In line with the vMDT, therapeutics was modified by 46 referees, which resulted in patient’s outcome improvement in 20, and no improvement in 24; at last follow-up, the treatment proposed by the vMDT had been modified by 20 respondents (due to limited efficacy or side effects). Referees requested deep brain stimulation recommendations in 13 cases; DBS was approved in 7 (including one case for who DBS discussion was not solicited by the referee) and performed in 6, with good results at last follow-up when the survey was filled.

### 3.5 Long-term follow up

**Figure 2:**
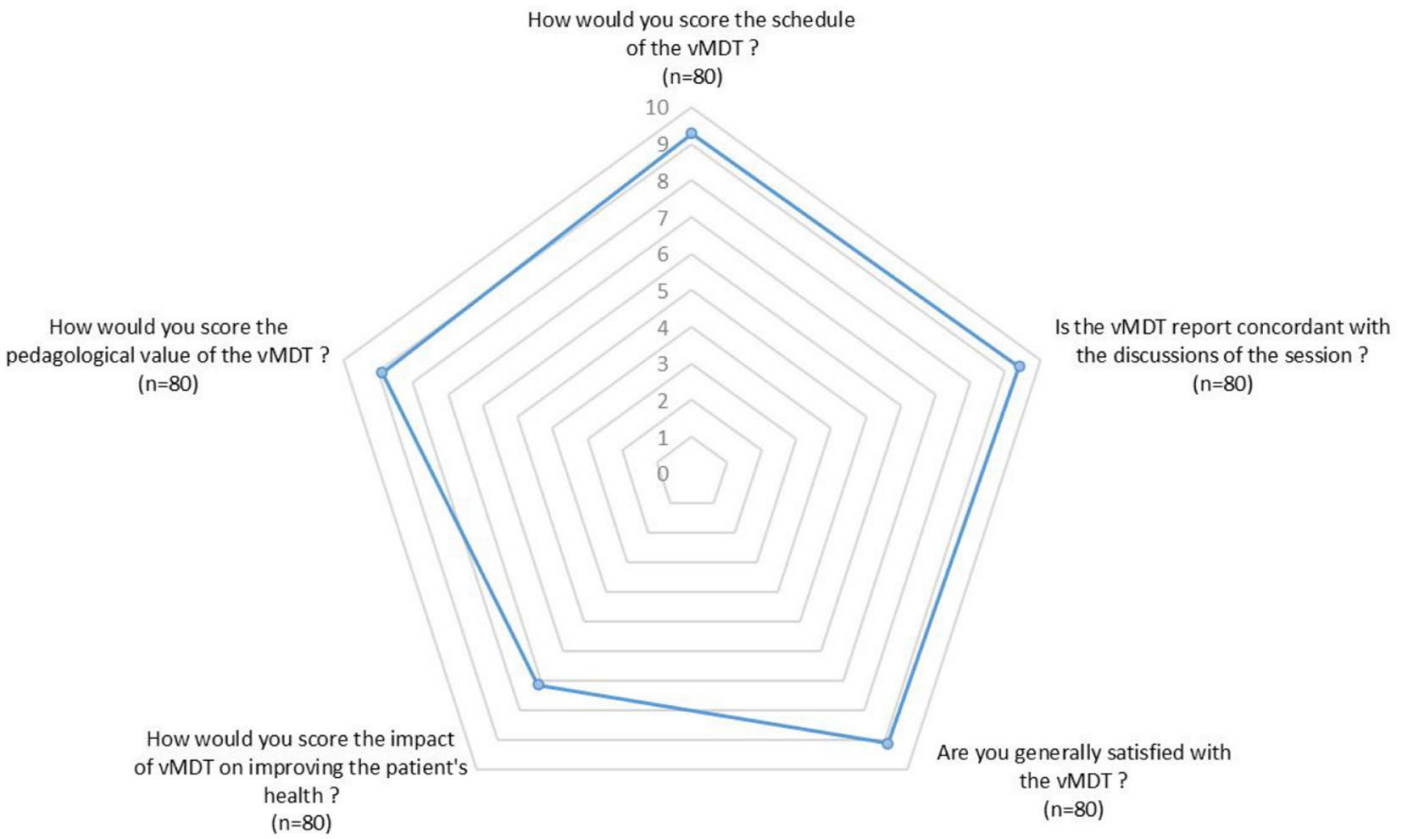
Radar chart illustrating the mean scores (min 0, max 10) given by respondents regarding different aspects of the vMDT (*n* = 80 respondents).

When they filled the questionnaire, 74 of the respondents were still in charge of the patient and 37 of them reported an overall outcome improvement 6 months after the vMDT. The respondent perceived the impact of the vMDT on this improvement with a mean score of 7.1/10 with 4 respondents scoring the impact of the vMDT below 5 (Figure 2). One patient died due to the natural course of his disease.

### 3.6 Overall satisfaction

Overall satisfaction among respondents was high, with a mean score of 9.1/10. Among the 83 respondents, 5 considered that the vMDT had little or no pedagogical interest (3 neuropediatricians, 1 adult neurologist and 1 pediatrician). On the other hand, 89% of the respondents stated that the vMDT contributed to improvement of their understanding and management of pediatric movement disorders; mean score for pedagogical value of the vMTD was 9/10 (Figure 2).

### 3.7 Opportunities for improvement

Some respondents pointed out the absence of follow-up and suggested follow-up sessions designed to share the outcomes of diagnostic and therapeutic recommendations.

### 3.8 Focus on the clinical cases’ content

The etiology of movement disorders was identified in 22/83 cases before the vMDT (27%); genetic diseases were diagnosed in 15/83 DYT11 (1 patient), Rett syndrome 2 patients; and pathogenic variants in the following gene:, *MED23* (1 patient), *GNAO1* (1 patient), *NALCN* (1 patient), 22q11 (1 patient), *POLR3A* (1 patient), *GABRB3* (1 patient), *ATP1A3* (2 patients), *PNKD* (1 patient), *ADCY5* (2 patient), *KMT2B* (1 patient); non-genetic cerebral palsy was diagnosed in 5/83 (kernicterus 1/83, neonatal anoxic ischemia 4/83), and inborn error of metabolism in 2/83 (methylmalonic aciduria and glucose transporter deficiency).

According to the respondents, movement disorders of submitted cases were categorized as isolated in 39 and combined in 44 and dystonia in 43/83 (52%), chorea–athetosis–ballismus in 19/83 (15%), dyskinesias in 22/83 (27%), tics in 4/83 (5%), parkinsonism in 1/83, tremor in 7/83 (8%), myoclonus in 9/83 (11%), stereotypies in 1% (1/83), abnormal ocular movements in 7/83 cases (8%) (Table 2). The vMDT modified the MD categorization in 33/83 cases (40%). Classification was revised by the vMDT in 8 cases (one case of each revised to: dyskinesia to tics; tics to obsessive compulsive behavior; tics to stereotypies; jerks to myoclonus; torticollis to abnormal posture secondary to gastro oesophageal reflux; dystonia to intense imagery movements; tremor to non-epileptic myoclonus; dystonia and ballism to functional neurological disorder). In 25 cases, the vMDT implemented the phenomenological categorization (identification of MD not identified by the respondent or more specific description of the MD, especially abnormal eye movements). Moreover, among the 19 cases with paroxysmal events, 4 were not classified in the isolated or combined groups, as vMDT established paroxysmal ataxia (2 cases), paroxysmal hypotonia (1 case) or paroxysmal hemiplegia (1 case).

**Table 2:** Categorization of the movement disorder type before the vMDT according to the submission form and after the vMDT according to the final report.

	Before PMD-vMDT	After PMD-vMDT
Movement disorders present	83	81
Including paroxysmal disorders	19	19
Combined movement disorders	44	42
Dystonia	21	29
Chorea/athetosis/ballism	15	15
Tremor	5	6
Myoclonus	6	7
Tics	1	1
Stereotypies	0	0
Parkinsonism	1	2
Dyskinesias	22	20
Abnormal ocular movements	3	3
Isolated movement disorders	39	35
Isolated abnormal ocular movements	4	5
Dystonia	22	17
Chorea/athetosis	4	3
Tremor	2	0
Myoclonus	3	5
Tics	3	2
Stereotypies	1	3
Parkinsonism	0	0

## 4 Discussion

Implementation of a virtual multidisciplinary team dedicated to pediatric movement disorder in 2018 was an innovative project in the domain of pediatric neurology practise. The increasing number of participants and the involvement of cross-borders colleagues highlighted the growing interest in this vMDT that rapidly spread among French-speaking pediatric neurologists. This trends toward vMDT have been clearly accelerated since the covid period; many vMDT have been developed and are now a gold standard in various medical specialties. Nevertheless, to our knowledge, there are few studies evaluating MDTs especially in the context of rare neurological diseases in childhood. As part of a professional practise evaluation, we decided to evaluate the PMD-vMDT, by exploring the experiences and perceptions of participants of the vMDT.

The results of our survey show that participants who submitted cases were satisfied with accessibility and ease of use of the vMDT, although 12/82 met difficulties to upload the videos. The vast majority of the participants highly rated the content of the discussion during the vMDT (interactivity, expertise, respect of the various opinions), and the quality of the final report.

Most of the case were referred to the vMDT for combined opinion regarding semiological/diagnosis management, or semiological/diagnosis plus therapeutic advice. The vMDT recommendations often extended beyond the request (therapeutic recommendation when only semiological opinion was requested, DBS proposal, diagnosis work-up recommendation when therapeutic recommendation request), and it is noteworthy that respondents massively reported that vMDT recommendations were implemented in the management of their patients. When focusing on cases requesting semiological recommendations, 50% of the categorisations were revised or implemented, and vMDT performed revision or implementation of MD classification in 40% of the whole cases. Such data are concordant with the Dutsch experience of the face-to-face multidisciplinary outpatient clinic that reported revision of movement disorder classification in 58/100 patients, with an increase in classification as dystonia and myoclonus, and decrease of ataxia and tremor (16). Dystonia was the most frequent MD among vMDT submitted cases, with modulation of its classification in almost a quarter of the cases after the session.

Most of the patients discussed during the vMDT are undiagnosed, and 80% of the respondents perceived that the conclusions and recommendations helped the etiological work-up. Actually, the vMDT is frequently questioned about genetic diagnosis, and how to conduct genetic tests (panel, exome sequencing, genome sequencing); although the survey did not collect data concerning these aspects, we can confirm that recommendations according to genetic guidelines are provided throughout the vMDT; it is noteworthy that genetic diagnosis proposed by the vMDT was confirmed in one third of the cases, although deep analysis of this data was not an objective or the survey. Semiological and diagnostic recommendations were perceived to improve the patient’s outcome by allowing to jump out of diagnosis uncertainty, and draw an action plan concerning investigation work-up and therapeutic strategy.

Positive impact on decision making and patient’s outcome is reported by the majority of the respondents (80% concerning semiological/diagnosis opinion). Despite adherence of the respondents to the vMDT recommendations, it must be highlighted that vMDT reports were not included in the patients’ file in 20% of the case, and information were shared with the families in only half of the cases. These points will have to be improved, as inclusion the report in the file and delivery of information to the families are mandatory according to the vMDT chart.

Although therapeutic recommendations were largely implemented by the vMDT, positive impact was reported in less than half of the patients. Proposed treatment was not efficient and /or at to be modified due to side effects in many patients. The partial improvement of patient’s outcome as perceived by the respondents may illustrate the well-known difficulties in treating childhood movement disorders (10, 11, 13) and may also highlight the limits of the benefits provided by the vMDT. The relative low score (as compared to the other ones) of « the impact of vMDT on improving the patients’ health » probably reflects limitations of the vMDT therapeutic recommendations as perceived by the respondents. Anyway, in the subgroup of patients who improved at follow-up, respondents established a clear link between health improvement and recommendation of the vMDT. The educational benefit of the vMDT has also been highlighted by the responders, who scored very nicely its pedagogic qualities, and who emphasised the enhancement of PMD expertise in their daily practise.

This study has some limitations, especially we could not go in deep semiological/diagnosis comparison before referral and after the vMDT; actually, the perception of the vMDT by the participants was targeted and follow-up data of the cases was not explored. Van Egmont’s study was focused in patients’ health and provided detailed information concerning benefit from a multidisciplinary team approach for PMD in terms of diagnosis and treatment in comparison to the referring specialists. After the survey, a follow-up section has been added to the vMDT submission forms, and “follow-up case discussion” have been encouraged. Prospective follow-up and data collection concerning diagnosis, treatment and outcome after vMDT will help to better delineate its real impact on PMD management; analysis of patients’ perception of care and of their health status after the vMDT may also be interesting.

Another limitation of this study is that it was conducted by the MDT coordinating team, and participants may have tailored their responses to be socially desirable.

Assessment of a case during vMDT relied on the description, neurological examination, and home or medical videos provided by the participant who submitted the case; clinical examination of children with movement disorders may be challenging for untrained practitioner; videos are crucial for movement disorders assessment, but their quality have an critical impact on interpretation of suspected MD and may lead to misdiagnosis (the video may not record a movement disorder that have not been recognized by the examiner for example). The limits of vMDT for PMD are intrinsically linked to its virtual concept (as it relies on the participant clinical examination, and on videos).

## 5 Conclusion

PMD-vMDT has been developed to ensure comprehensive decision-making, to overcome geographical barriers (illustrated by participants from many foreign and far away countries), and to ensure an equal access to expertise. Technological issues or lack of skills to navigate technology have been enhanced in some surveys (9); the easy and secured platforms used for the PMD-vMTD were rather considered as technologically friendly by the respondents. The very high overall satisfaction encourages us to continue this vMDT, and while being mindful of its inherent limitations, the result of this survey may promote the development of other PMD-vMDT over the world.

## Data Availability

The original contributions presented in the study are included in the article/Supplementary material, further inquiries can be directed to the corresponding author.

## Group members of the PMD-vMDT group

Abi Warde Marie Thérèse, Neurologie pédiatrique CHU de Strasbourg, Centre de Référence Constitutif Carammel, Centre de Compétence Maladies Héréditaires du Métabolisme, Starsbourg, France, marie-therese.abi-warde@chru-strasbourg.fr; Altenburger Lucile, Département des maladies chroniques de l’Enfant, Hôpital Universitaire de Nantes, Nantes, France, lucile.altenburger@chu-nantes.fr; Biboulet Bruneau Céline, Service de pédiatrie de spécialités CHU Grenoble Hôpital Couple Enfants, Grenoble, CBruneau@chu-grenoble.fr; Bonheur Julie, DYSPA Unit, Pediatric Neurosurgery Department, Rare Disease Competence Center Neurogene, Rothschild Foundation Hospital, Paris, France, jbonheur@for.paris; Bommier Laur Domitille, DYSPA Unit, Pediatric Neurosurgery Department, Rare Disease Competence Center Neurogene, Rothschild Foundation Hospital, Paris, France, dbommierlaur@for.paris; Ians-Bouteiller Cécile Ians-Bouteiller, Department of Pédiatric Neurology and Rehabilitation, Bordeaux University Hospital, Bordeaux, France, cecile.bouteiller@chu-bordeaux.fr; Bouchereau juliette, Centre de référence des maladies héréditaires du métabolisme—Hôpital Necker Enfants Malade—APHP-Paris, Filière des maladies métaboliques G2M, France, Juliette.bouchereau@aphp.fr; Burglen Lydie, Centre de référence des malformations et maladies congénitales du cervelet et Laboratoire de neurogénétique pédiatrique, Département de génétique, APHP, Sorbonne Université, Hôpital Armand Trousseau, Paris, France, lydie.burglen@aphp.fr; Buzatu Marga, Neurologie Pédiatrique, CHU Charleroi, Belgique, marga.buzatu@humani.be; Cabasson Sébastien, Service de neuropédiatrie et Soins intensifs pédiatriques, Hôpital de Paul, France, sebastien.cabasson@ch-pau.fr; Chevassus Hugues, Centre d’Investigation Clinique, CHU de Montpellier, CIC1411, Inserm, Montpellier, France, h-chevassus@chu-montpellier.fr; Chouchane Mondher, Consultation de neurologie pédiatrique, service de pédiatrie 1, hôpital d’enfants, CHU Dijon Bourgogne, université Bourgogne Europe, Dijon, France, mondher.chouchane@chu-dijon.fr; Cleuziou Pierre, Centre Hospitalier Universitaire de Lille, Clinique de Pédiatrie, Service de Neuropédiatrie, Lille, France, pierre.cleuziou@chu-lille.fr; Coget Arthur, Neuroradiology Department, University Hospital, Montpellier, France, arthur-coget@chu-montpellier.fr; Dabudyk Thibaud, Réanimation infantile, Centre Hospitalo-Universitaire de Besançon, Université de Franche-Comté, Besançon, France, tdabudyk@chu-besancon.fr; Damaj Lena, Centres de Compétences maladies héréditaires du métabolisme, et leukodystrophies et leuco encéphalopathies rares, Service de Génétique Clinique, CHU Rennes, lena.damaj@chu-rennes.fr`; de Lattre Capucine, ESEAN APF France Handicap, SMR pédiatrique, Nantes, capucine.delattre@apf.asso.fr; Dorison Nathalie, DYSPA Unit, Pediatric Neurosurgery Department, Rare Disease Competence Center Neurogene, Rothschild Foundation Hospital, Paris, France, ndorison@for.paris; Doummar Diane, APHP Sorbonne Université, Service de Neurologie Pédiatrique, Centre de Référence Neurogénétique, Hôpital Armand Trousseau, Paris, France, diane.doummar@aphp.fr; Dubois fanny, Service de pédiatrie de spécialités CHU Grenoble Hôpital Couple Enfants, Grenoble, FDubois@chu-grenoble.fr; Gins Clarisse, Service de pédiatrie, GHBS Lorient, Bretagne Sud, Lorient, France, c.gins@ghbs.bzh; Heidy Barde Mairena, Service de Neuropédiatrie, CHU Gui de Chauliac, Montpellier, France, heidy.baidemairena@chu-montpellier.fr; Gras Domitille, CRTLA service neuropédiatrie CHU Bicêtre, APHP; Sorbonne University, Inserm U1127, CNRS UMR7225, Paris Brain Institute, Paris, France; CEA Paris-Saclay, Frederic Joliot Institute, NeuroSpin, UNIACT, Gif-sur-Yvette, France, domitille.gras@aphp.fr; Hully Marie, Service de Neuropédiatrie, CHU Necker Enfants Malades, Paris, France, marie.hully@aphp.fr; Khabbach Kaoutar, Faculté de medecine et de pharmacie de Tanger, université Abdelmalek Essaâdi Maroc. Centre hospitalier universitaire Mohammed VI de Tanger, Maroc, kawtarkhabbach@gmail.com; Loussouarn Anna, Neurologie pédiatrique, AP-HP Université Paris Saclay, Hôpital Bicêtre. Centre de référence des maladies héréditaires du métabolisme, Paris, France, anna.loussouarn@aphp.fr; Leboucq Nicolas, Neuroradiology Department, University Hospital, Montpellier, France, n-leboucq@chu-montpellier.fr; Lion-François Laurence, Service de Neuropédiatrie, Hôpital Femme Mère Enfant, Hospices Civils de Lyon, Bron, France, laurence.lion-francois@chu-lyon.fr; Mignot Cyril, APHP Sorbonne Université, Département de Génétique, Groupe Hospitalier Pitié-Salpêtrière-hôpital Trousseau, Paris, France, cyril.mignot@aphp.fr; More Rebecca, Department of Pediatric Neurology, Charles Nicolle University Hospital, Rouen, France, rebecca.more@chu-rouen.fr; Pacteau Chloé, Service de MPR pédiatrique, CHU de Saint Etienne, France, chloe.pacteau@gmail.com; Panagiotakaki Eleni, Department of Paediatric Clinical Epileptology, Sleep Disorders and Functional Neurology, Member of the ERN EpiCARE, University Hospital of Lyon (HCL), Lyon Neuroscience Research Center, Inserm U1028/CNRS UMR 5292, Lyon, France, eleni.panagiotakaki@chu-lyon.fr; Ravelli Claudia, APHP Sorbonne Université, Service de Neurologie Pédiatrique, Centre de Référence Neurogénétique, Hôpital Armand Trousseau, Paris, France, claudia.ravelli@aphp.fr; Riant Florence, AP-HP Sorbonne Université, Département de génétique médicale, UF de Neurogénétique Moléculaire et Cellulaire, Hôpital Pitié-Salpêtrière, Paris, France, florence.riant@aphp.fr; Rivier Ringenbach Clotilde, Service de Pédiatrie, CH Nord Ouest, Villefranche, France, crivier@HNO.fr; Roze Emmanuel, Sorbonne Université, Institut du Cerveau, INSERM, CNRS, AP-HP, Hôpital de la Salpêtrière, Intitut de Neurologie, Paris, France, emmanuel.flamand-roze@aphp.fr; Roujeau Thomas, Service de neurochirurgie pédiatrique, CHU Montpellier, Montpellier France, t-roujeau@chu-montpellier.fr; Sarda Eugénie, Service de Neuropédiatrie, CHU Necker Enfants Malades, Paris, France, eugenie.sarda@aphp.fr; Sarret Catherine, Pédiatrie et Génétique Médicale, Hôpital Estaing, CHU Clermont-Ferrand, Clermont-Ferrand, France, csarret@chu-clermontferrand.fr; Todosi Calina, Service de Pédiatrie, CHU Nancy, Nancy France, c.todosi@chru-nancy.fr; Vayssiere Pia, DYSPA Unit, Pediatric Neurosurgery Department, Rare Disease Competence Center Neurogene, Rothschild Foundation Hospital, Paris, France, pvayssiere@for.paris; Yapo Apo Constance, Unité pédagogique système nerveux-réadaptation, Abidjan, Cpote d’Ivoire, apo.constanceyapo@gmail.com; Yazbeck Elise, Service de Neuropédiatrie, CHU de Bicêtre, Université Paris-Saclay, Paris, France, elise.yazbeck@aphp.fr.

## Supplementary material

SUPPLEMENTARY MATERIAL 1 Survey questionnaire.
