# Supplementary material for: French virtual multidisciplinary team meeting for pediatric movement disorders (PMD-vMDT): a three-year survey
Source: Front Neurol. 2026 Jan 13;16:1751665. doi: 10.3389/fneur.2025.1751665 (PMC12834823; doi:10.3389/fneur.2025.1751665)
Supplement: SUPPLEMENTARY MATERIAL 1 — Survey questionnaire. [file Data_Sheet_1.pdf]

The questionnaire collected objective and subjective data on professionals' experience using 46 items divided into five sections including pre-vMDT data, vMDT outcome, post- MDT impact, long term follow up and overall satisfaction. It included single-choice items, satisfaction scales (from 1 to 10), and open-ended questions. It included single-choice items, satisfaction scales (from 1 to 10), and open-ended questions.

The first section explored the preliminary steps before the meeting, especially information given to families about the vMDT and the sharing of clinical videos, as well as the perceived ease of submitting the case file (via standardized form and uploading documents to a secure drive). Respondents were then asked to indicate the types of disorders presented by the patient according to established classification (15) (dystonia, chorea, dyskinesia, gait disorders, others) and to specify the nature of their request (semiological opinion, diagnostic advice, therapeutic recommendation, deep brain stimulation recommendations).

The second section focused on the organisation of the vMDT : adherence to the schedule, participants' panel, quality of the discussion, interactions regarding the case presented, and perception of the consideration given to individual remarks. The degree of consensus around the final decision was also assessed, as well as the perceived quality of the argumentation (based on guidelines, clinical experience, or bibliography). Particular attention was paid to the accuracy of the transmitted report, its clarity, and its conformity with the discussions.

The third section addressed the post-vMDT impact, with 3 optional subsections depending on the initial request (semiological opinion, diagnostic opinion, therapeutic advice, in particular deep brain stimulation). For semiological opinions, the questionnaire explored whether the diagnosis proposed was different from the one initially considered, whether it helped to guide the etiological investigation, and whether the conclusions were integrated into the medical file. For diagnostic and therapeutic opinions, respondents were asked to indicate if the vMDT had contributed to improving patient's management, led to adaptation or modification of treatments, avoided unnecessary investigations, or provided more accurate information for the families. In the case of DBS discussion, the questionnaire evaluated the actual implementation of the procedure, perceived effectiveness, and clinical impact.

The section 4 documented long-term follow-up: patient status (improvement, stability, deterioration, death, lost to follow-up), and perception of the direct influence of the vMDT on the course of the disease.

Finally, a section on overall satisfaction allowed respondents to evaluate the usefulness of the vMDT in their practice, the general quality of the system, as well as its contributions in terms of understanding and managing movement disorders in children. Respondents could also freely provide suggestions for improvement.

Evaluation de la RCP-PME

Afin de promouvoir la RCP-PME, nous souhaiterions connaître l'impact de la RCP sur le suivi des dossiers patients dont nous avons discuté ensemble. Nous aimerions également avoir votre avis sur le déroulement de la RCP.
Merci d'avance pour vos retours.
Agathe et Bérénice
En collaboration avec le Dr Jean-Gilles Rodier, médecin responsable qualité

* Obligatoire

Questions générales Pré-RCP

1. Avez-vous informé le patient et/ou sa famille sur la tenue de la RCP ? *

◯ Oui

◯ Non

2. Avez-vous informé le patient et/ou sa famille sur le partage de vidéo ? *

◯ Oui

◯ Non

3. Concernant de dépôt d'un dossier, comment avez-vous trouvé : *

	Très compliqué	Compliqué	Pas si simple	Simple	Très simple
La demande de présentation (accès à la RCP)	◯	◯	◯	◯	◯
La fiche RCP à remplir	◯	◯	◯	◯	◯
Le dépôt des documents sur le drive	◯	◯	◯	◯	◯

4. Êtes-vous satisfait du délai de la tenue de la RCP ? *

0	1	2	3	4	5	6	7	8	9	10

Pas satisfait — Très satisfait

5. Pour quelle(s) pathologie(s) avez-vous demandé la RCP ? *

- ☐ Dystonies
- ☐ Chorées
- ☐ Dyskinésies
- ☐ Syndrome Parkinsonien
- ☐ Troubles de la marche
- ☐ Tics
- ☐ Autres

6. Pour quel(s) motif(s) avez-vous demandé la RCP ? *

- ☐ Avis Sémiologique
- ☐ Avis Diagnostique
- ☐ Avis Thérapeutique
- ☐ Aide IRM
- ☐ Dossier DBS

7. Avez-vous sollicité un avis direct à une autre équipe ou à une autre RCP avant la RCP-PME ? *

- ○ Oui
- ○ Non

Questions générales concernant le déroulement de la RCP

8. La RCP s'est-elle tenue à la date programmée ? *

- Oui
- Non

9. Le dossier a-t-il été discuté par des spécialistes adaptés à votre question ? *

- Oui
- Non

10. Comment estimez vous la qualité de : *

	Pas satisfaisante	Peu satisfaisante	Plutôt satisfaisante	Satisfaisante	Très satisfaisante
Le discussion autour de votre dossier	◯	◯	◯	◯	◯
L'interactivité de la discussion autour de votre dossier	◯	◯	◯	◯	◯

11. Votre avis, opinion, vos remarques ont-ils été pris en compte ? *

- Oui
- Non

12. La décision a-t-elle fait l'objet d'un consensus ? *

- Oui
- Non

13. La décision vous a-t-elle paru argumentée ? (Expérience, référentiel, bibliographie) *

- Oui
- Non

Questions générales post-RCP

14. Le compte-rendu de la RCP est-il fidèle aux discussions ? *

0	1	2	3	4	5	6	7	8	9	10

Très éloigné — Tout à fait fidèle

15. Le compte-rendu vous a-t-il paru discutable ? *

- ◯ Oui
- ◯ Non

16. Avez-vous informé le patient et/ou la famille du compte-rendu de la RCP ? *

- ◯ Oui
- ◯ Non

17. Avez-vous recopier la conclusion de la RCP dans le dossier du patient ? *

- ◯ Oui
- ◯ Non

18. Avez-vous joint la fiche compte-rendu au dossier du patient ? *

- ◯ Oui
- ◯ Non

Questions Spécifiques "Avis sémiologique" post-RCP

A ne remplir que si un avis sémiologique a été demandé à la RCP

19. L'avis sémiologique est-il différent de celui que vous aviez évoqué ?

- ◯ Oui
- ◯ Non
- ◯ A apporté des précisions

20. Cet avis a-t-il permis d'orienter l'enquête étiologique ?

- ◯ Oui
- ◯ Non

21. Pensez-vous que la RCP vous ait permis d'améliorer l'état de santé du patient ? (adapter le traitement, éviter ou orienter les examens complémentaires, donner une information plus précise à la famille)

- ◯ Oui
- ◯ Non

22. Avez-vous suivi les indications/préconisations de la RCP ?

- ◯ Oui
- ◯ Non

Questions spécifiques "Avis diagnostic" post-RCP

A ne remplir que si un avis diagnostic a été demandé à la RCP

23. Pensez-vous que la RCP vous ait aidé pour la prise en charge thérapeutique du patient ?

- Oui
- Non

24. Pensez-vous que la RCP vous ait permis d'améliorer la prise en charge diagnostic du patient ?

- Oui
- Non

25. Pensez-vous que la RCP vous ait permis d'améliorer l'état de santé du patient ?

- Oui
- Non
- Ne sait pas

26. Avez-vous suivi les indications/préconisations de la RCP ?

- Oui
- Non

27. Un diagnostic génétique a-t-il été évoqué à la RCP ?

- Oui
- Non

28. Si oui, le diagnostic génétique a-t-il été confirmé ?

- Oui
- Non
- En cours

Questions spécifiques "Avis thérapeutique" post-RCP

A ne remplir que si un avis thérapeutique a été demandé à la RCP

29. Pensez-vous que la RCP vous ait aidé pour la prise en charge thérapeutique du patient ?

- ◯ Oui
- ◯ Non

30. Avez-vous changé le traitement du patient selon la RCP ?

- ◯ Oui
- ◯ Non

31. Pensez-vous que le traitement mis en place selon les recommandation de la RCP vous ait permis d'améliorer l'état de santé du patient ?

- ◯ Oui
- ◯ Non

32. Avez-vous modifié le traitement, du fait de l'inefficacité ou de la mauvaise tolérance ?

- ◯ Oui
- ◯ Non

33. Avez-vous suivi les indications/préconisations de la RCP ?

- ◯ Oui
- ◯ Non

Questions spécifiques "avis DBS" post-RCP

A ne remplir que si un avis DBS a été demandé à la RCP

34. Une DBS a-t-elle été retenue lors de la RCP ?

◯ Oui

◯ Non

35. Combien de temps après la RCP, la DBS a-t-elle été mise en place ?

	1 mois	1 à 3 mois	3 à 6 mois	6 mois à 1 an	plus de 1 an
Affirmation 1	◯	◯	◯	◯	◯

36. La DBS a-t-elle été efficace ?

◯ Oui

◯ Non

Suivi de l'enfant

37. Date des dernières nouvelles du patient *

38. Le patient est-il perdu de vue ? *

- Oui
- Non

39. L'état du patient s'est-il amélioré ? *

- Oui
- Non

40. Si oui, quel a été l'impact de la RCP ?

0	1	2	3	4	5	6	7	8	9	10

Pas efficace — Très efficace

41. Le patient est-il décédé ? *

- Oui
- Non

42. Si oui, date du décès :

43. Si oui, cause du décès (liée à l'évolution de la maladie, liée à une complication de la maladie, cause différente de la maladie suivie...) :

Satisfaction globale

44. Êtes-vous globalement satisfait de la RCP ? *

0	1	2	3	4	5	6	7	8	9	10

Pas satisfait — Très satisfait

45. Pensez-vous que la RCP vous ait permis d'améliorer votre compréhension dans la prise en charge des pathologies du mouvement de l'enfant ? *

- ◯ Oui
- ◯ Non

46. Avez-vous des suggestions pour améliorer la qualité de la RCP ? *

Ce contenu n’a pas été créé ni n’est approuvé par Microsoft. Les données que vous soumettez sont envoyées au propriétaire du formulaire.

Microsoft Forms
